# “Psychometric properties of the Arabic version of the post-traumatic growth inventory with university students in Jordan

**DOI:** 10.1016/j.heliyon.2023.e14211

**Published:** 2023-03-09

**Authors:** Mais Al-Nasa'h, Kimberly Asner-Self, Hassan Al Omari, Amani Qashmer, Mohammad Alkhawaldeh

**Affiliations:** a Department of Counseling and Special Education, School of Educational Sciences, The University of Jordan, Jordan; b Clinical Mental Health Program, The School of Health Sciences, Touro University, USA; c Department of Educational Psychology, School of Educational Sciences, The University of Jordan, Jordan

**Keywords:** Post-traumatic growth, Arabic translation, Scale validation, Growth inventory, Psychometric properties

## Abstract

Post-traumatic Growth plays a key role to cope with traumatic incidents. The scale for Post-Traumatic Growth Inventory (PTGI) has been used by several researchers in different languages. This study aims to evaluate the Arabic-translated version of the PTGI scale by focusing on its validity in different languages and contexts. This study introduces an Arabic version of the PTGI-M normed with 417 undergraduate students at a large university in Jordan. The internal consistency (Cronbach's alpha) and test-retest reliability of the instrument were 0.97 and 0.82, respectively. Bivariate correlation was used to approximate the concurrent validity (CV). Significant correlations were found between the PTGI-M and the beck depression inventory (BDI), perceived stress scale (PSS), Taylor's manifest anxiety (TMAS), satisfaction with life (SWL), and the Rosenberg self-esteem scale (RSES). Confirmatory factor analysis (CFA) to assess the convergent and discriminant validity of the translated scale. Convergent and discriminant validity was established for the Arabic version of the PTGI-M by conducting a confirmatory factor analysis (CFA). In conclusion, this study proposes that future investigations should consider analysing the total PTGI-M subtotal scores to comprehend the complexity of the post-traumatic growth experience.

## 1 Introduction

Recent evidence suggests that one of the costs of life is experiencing or witnessing potentially traumatising events, such as death (actual, threatened, violent, and unexpected), violent crime, sexual violence, domestic violence, serious injury, accidents, natural disasters, human-made disasters (for example, chemical spills, nuclear plant failure), war, and civil unrest (troops and civilians) [1]. The World Health Organisation (WHO) [2] estimates the trauma lifetime prevalence rate worldwide as 71% with 3.2-lifetime exposures per capita [3]. Moreover, exposure to trauma is often associated with different types of psychological stress such as “anxiety, depression, and post-traumatic stress disorder” (PTSD). The severity of distress could be influenced by the type of trauma [1,4]. People find themselves grappling with the loss of the “old normal” and the integration of the “new normal.” To date, humans have survived and thrived after traumatic events. Since the 1980s, research into post-trauma thriving, resilience, and hardiness, suggests that as people grapple with their grief and pain, they begin to thresh out positive changes in their lives [[5], [6], [7], [8]].

The post-traumatic growth inventory (PTGI) has been reported as a key driver to address negative consequences and boost self-esteem after facing traumatic events [9]. Previous studies demonstrated that PTG enabled the person to mitigate depression, stress, anxiety and other negative psychological factors [[10], [11], [12]]. A recent study defined “PTG as where someone has been affected by PTSD and finds a way to take new meaning from their experiences to live their lives differently than before the trauma” [12]. This PTG assists people to improve their life satisfaction and self-esteem levels. Literature supports this argument, as individuals with enriched PTGI are more capable to handle stress, depression, and anxiety as compared to those with poor PTGI [13,14].

“Post-Traumatic Growth (PTG)” is known as post-trauma positive psychological and personal development [8]. Though several inventories have been developed to evaluate PTG, PTGI is the most studied psychometrically and used empirically both in the United States and internationally with myriad populations. It is pertinent to recognise the requirement for a culturally sensitive scale that is valid in investigating PTG. From a cultural viewpoint, performing a review would be advantageous in identifying the importance of oneself and the awareness of others as cultural beings. As a rich construct, PTG and its experience differ from a cultural context, which is important in comprehending the expression of PTG in distinct environments [15].

### 1.1 The rationale for the current study

As a developing country, young people aged between 18 and 34 years old account for approximately 40% of Jordan's population [16]. The present number of university students in Jordan is estimated at 300,000, representing 4% of the nation's population. Concurrently, there is an increasing trend in the incidence of traumatic events given the current lifestyles of the younger generation. However, despite the plethora of literature on PTG targeting older populations from distinct cultures and heritage, limited studies have examined PTG in eastern ethnicities [17]. A recent study on PTGI that was translated into Arabic focused on exploring and validating PTGI events of orphans' population experiencing political upheaval and conflict [18]. This event is similar to the situation in the Gaza strip and the armed conflict due to political unrest in the country. Authors in Ref. [19] also employed a snowball sampling method in selecting a small sample but failed to provide a good indication of the overall population.

Given the discussion above suggesting that PTGI was initially thought to be normal regarding university students [8], and contemplating the political and cultural disparities between the Gaza strip and the Hashemite Kingdom of Jordan, this study aims to (a) review and verify a culturally and psychometrically version (in Arabic) of PTGI using a non-probability sampling, and (2) to examine the rise of PTG among university students in Jordan.

The original scale developed by authors in Ref. [8], focused on a group of undergraduate students who had been hired from psychology classes at a prominent university in the southeastern United States. In the study, the English version of the PTGI scale was primarily designed based on the responses from students. Meanwhile, the other Arabic scales for PTGI were translated by Ref. [19] among Palestinian adults, by Ref. [20] on Middle Eastern refugees in Australia, and the Arabic version developed by Ref. [21]. Specifically, authors in Ref. [20] employed an EFA in their study to explore the factors for PTGI.

All the aforementioned scales utilised different samples, including refugees, adults, and participants from other demographics. In contrast, the present study focused on students with identical characteristics to those used in the original scale in assessing how the respondents reply to the Arabic version of the PTGI scale. Thus, the novelty of this study is the approach employed in the Arabic version scale involving the same sample of students as those in the original scale development. Table 1 depicts the inconsistencies in the translation of items for this scale due to the grammatical and contextual differences in Arabic regional languages. This study contributes to the existing literature by pre-testing the newly translated Arabic version to achieve content validity. Furthermore, convergent and discriminant validity for the translated scale was also performed using the CFA. The inconsistent findings from the previously translated scales of various studies also highlight the issues on the reliability and validity of the scale. The present study attempts to mitigate these issues by performing a back-to-back translation.

**Table 1:** Interpretations of the differences between the meaning of each of the items that differ, and reasons for choosing the items reported in this study.

PTGI-K PTGI-M PTGI-T PTGI-Original				#
*غيرت اولوياتي حول ماهو مهم في الحياة*	لقد غيّرت اولوياتي حسب أهميتها في حياتي	تغيرت أهدافي في الحياة بعد الحرب مقارنة لما هي عليه قبل الحرب	I changed my priorities about what is important in life	1
*ازداد تقديري لنفسي .*	أصبح لدي امتنان وتقدير أكثر لقيمة حياتي	اقدر قيمة حياتي أكثر من الأول	I have a greater appreciation for the value of my own life.	2
*كونت اهتمامات جديدة*	لقد طورت اهتمامات جديدة	بدأت اهتم بأشياء جديدة في الحياة	I developed new interests.	3
*ازداد شعوري بالاعتماد على النفس.*	لدي شعور اكبر بأني اعتمد على نفسي	أصبحت ثقتي في نفسي أكثر من قبل	I have a greater feeling of self-reliance.	4
*اصبح لدى فهم افضل للأمور الروحية.*	.أصبح لديَّ فهم أفضل للنواحي الروحانية	أصبحت أتفهم الأمور الروحية و الدينية أفضل من قبل	I have a better understanding of spiritual matters	5
*أصبحت اكثر قدرة على الاعتماد على الناس في وقت الشدة.*	استطيع أن ارى بوضوح اكثر انه بامكاني الاعتماد على الاخرين في الاوقات الصعبة	عرفت بأنني استطيع الاعتماد على الآخرين حولي عندما أقع في مشكلة	I more clearly see that I can count on people in times of trouble	6
*شقيت طرق جديدة لحياتي .*	لقد قمت بتأسيس طريقة جديدة لاعيش حياتي	اخترت طريق (مسار) جديد في حياتي	I established a new path for my life.	7
*أصبحت اشعر آني اكثر ارتباطا بالآخرين.*	.أصبح لدي شعور اني اكثر قرباً من الاخرين	أشعر بالقرب من الآخرين	I have a greater sense of closeness with others.	8
*أصبحت اشد استعدادا للتعبير عن انفعالاتي.*	لدي استعداد اكبر لاعبّر عن مشاعري	أصبحت قادرا على التعبير عن مشاعري أكثر من قبل الحرب	I am more willing to express my emotions.	9
*زادت قدرتي في مواجهة الصعوبات.*	أصبحت لدي قدرة أكبر في مواجهة الصعاب	أعرف بأنني أصبحت قادرا بطريقة أفضل على التعامل مع مشاكلي	I know better that I can handle difficulties.	10
*أصبحت اكثر قدرة علي إنجاز أعمال جيدة في حياتي*	لدي القدرة لعمل أشياء أفضل في حياتي	أستطيع أن أفعل الأشياء في حياتي بطريقة جيدة بعد الحرب	I am able to do better things with my life.	11
*أصبحت اكثر قدرة على تقبل الواقع.*	أستطيع بشكل افضل أن أتقبل الطريقة التي تجري بها الحياة	أقبل بشكل أفضل ما انتهت إليه الأمور بعد الحرب	I am better able to accept the way things work out	12
*ازداد تقديري لحياتي يوم بعد يوم.*	.أستطيع أن اكون ممتن بشكل اكبر لحياتي يوماً بعد يوم	اقدر كل يوم جديد في حياتي أكثر من الأول	I can better appreciate each day	13
*أصبحت هناك فرص جديدة متاحة, لم تكن متاحة من قبل.*	أصح هنالك العديد من الفرص المتوفرة والتي لم تكن متاحة قبل ذلك	أصبحت لدي فرص جديدة في الحياة لم تكن موجودة من قبل	New opportunities are available which wouldn't have been otherwise	14
*أصبحت اكثر إحساسا بالآخرين.*	لديّ تعاطف اكثر مع الاخرين	أصبحت لدي عاطفة و حب تجاه الآخرين	I have more compassion for others.	15
*ازدادت جهودي لتكوين علاقات مع الآخرين.*	إنني أبذل جهود أكبر ضمن علاقاتي الشخصية	أحاول أن أقيم أفضل العلاقات الاجتماعية مع الآخرين	I put more effort into my relationships.	16
*أصبحت اكثر استعدادا لتغير الأوضاع التي تحتاج إلى تغيير.*	لديّ رغبة أكبر لمحاولة تغيير الاشياء التي بحاجة الى ان تتغير	. أحاول أن أغير الأشياء في الحياة التي تحتاج للتغيير	I am more likely to try to change things which need changing	17
*قوى إيماني الديني.*	أصبح لديّ ايمان اقوى بديني	أصبح أيماني أعمق بالله	I have a stronger religious faith	18
*اكتشفت انني اشد قوة مما كنت اتصور.*	.لقد تبين لي انني اقوى مما كنت اتوقع	اكتشفت بأنني أكثر قوة مما كنت أعتقد	I discovered that I'm stronger than I thought I was.	19
*أهم درس تعلمته هو" أن الناس رائعين"*	لقد تعلّمت درس هام كيف أن الناس رائعون	تعلمت كثيراً كيف أن الناس حولي رائعين	I learned a great deal about how wonderful people are.	20
*ازداد تقبلي لمبدأ احتياجي للاخرين*	.أصبحت اتقبل بشكل أفضل مبدأ اني قد أحتاج للاخرين	*تقبلت أكثر من قبل بأنني أحتاج الناس من حولي*	I better accept needing others	21

PTGI-K PTGI-M PTGI-T PTGI-Original#.

## 2 Material and methods

### 2.1 Participants and study procedure

This study was conducted among undergraduate educational science students at the University of Jordan who were recruited to complete a paper-and-pencil survey packet. The study was authorised by the “Institutional Review Board (IRB)”. Participation was voluntary and no additional credit was awarded to students. Meanwhile, the students were free to terminate their participation and withdraw from the study with no consequence or penalty. A total of 446 undergraduate students enrolled in educational sciences courses at a large university in Jordan were selected. Each sampled student was asked a screening question before data collection. Students that were yet to experience trauma in their recent past were not enrolled on the study. Resultantly, 29 students (six males and 23 females) who were identified to have never experienced trauma were excluded from the study before data analysis. Furthermore, data from 11 of the 29 students were considered unsuitable for further analysis since at least nine items from the PTGI-M were missing for these subjects. Hence, the dataset from 417 students was subjected to confirmatory factor analysis.

Students were first briefed on the research background and objectives. Subsequently, the participants were given a 40-min survey to complete during class. A student volunteer collected the surveys once completed and transferred the filled forms to the recruiter (researcher), who passed these on to the first and third authors. To ensure the test-retest reliability, 50 students were selected as participants for this longitudinal survey design. These selected students were assigned an anonymous student ID to identify them for the second phase of pilot testing for reliability purposes. The first and second tests were conducted at two-week intervals. This study utilised the students’ IDs in collecting data from the same respondents. The whole process was approved by the Institutional Review Board (IRB) of the university. Upon completing the tests, the answers were matched and evaluated based on the given student IDs.

Ensuring common method bias is also important, because it can lead to inaccurate conclusions and invalidate research findings. Common method bias occurs when a single method of data collection, such as a survey, introduces systematic error into the results by measuring multiple constructs, and the results for those constructs are correlated in an unexpected way. Few steps were taken to control common method bias (CBM). Firstly, this study ensured the anonymity and confidentiality of respondents. Secondly, the questionnaire was designed by shuffling the constructs, which made it impossible for respondents to differentiate between dependent and independent constructs [22]. This study ensured CBM by using multi-collinearity in the context of SEM (structural equation modelling) as described in Ref. [23]. Variance inflation factors (VIF) values were also employed to identify and ensure CBM. If the values of VIF in collinearity are higher than 3.3 indicates CBM presence in the model. In this study, all VIF values were less than 3.3, which corroborated that the model of this study is not contaminated by CBM. Thus, this study was free of CMV. Smart-PLS 3 was also used for the data analysis.

### 2.2 The post-traumatic growth inventory

The PTGI is an instrument comprising 21 items designed to estimate the construct of “post-traumatic growth” or personal-positive growth after life-crisis experiences [8]. Normed on undergraduate students in the US, the scale encompasses five factors: “New Possibilities (five items), Relating to others (seven items), Personal Strength (four items), Appreciation of Life (three items), and Spiritual Change (two items)”. Agreement with each item was indicated by using a six-point Likert scale ranging from 0 = not experienced this change to 5 = experienced this change to a high degree. The five sub-factors were summed up to obtain the overall PTGI score with higher scores correlating with greater perceived growth [24,25]. Studies in the US consistently indicate strong internal reliability for the total score (Cronbach's alpha: α = 0.90), moderate to strong reliability for the subscales, and adequate test-retest reliability over two months (α = 0.71) [[26], [27], [28]]. Conflicting findings were reported regarding the support of the five-factor model proposed by authors in Ref. [8]. In the literature review, a consistent pattern was observed with high subscale intercorrelations suggesting alternative one, two, and three-factor models might be more indicative of the overall construct of the PTGI.

The PTGI has been translated and used in European countries, including refugees in Bosnia [29], cancer patients in the Netherlands [30], stroke victims in Germany [31], and general population in Turkey and Spain [32]. In the Asian context, the translated PTGI has been applied among Chinese cancer patients [33], and those with chronic diseases [34], as well as Japanese university students [35]. Similarly, the Arabic version of the PTGI has been translated and used among Arabic-speaking populations [19,20]. The present study refers to the Arabic versions of the PTGI as PTGI-K, PTGI-T, and PTGI-M as translated and studied by Refs. [19,21], and Authors (2023), respectively.

Counselling is now a global phenomenon; hence, culturally appropriate psychological assessment is necessary to evaluate human behaviours. This requires that measures are not only psychometrically sound [36] but are also culturally meaningful [[37], [38], [39]] Exact word-for-word translation rarely results in conveying the communication essence. For example, the phrase “I feel blue” translates directly into “انا اشعرازرق”, which does not mean anything. What is meant, however, is “I feel sad or depressed.” Should a bilingual counsellor who understands the contextual meaning of the phrase translates “I feel blue” to “انا أشعر بالاكتئاب”, the item may be measuring what we want it to measure. For further checking, another bilingual counsellor needs to back translate the phrase into English, and the phrase becomes “I am depressed”. This latter translation is not an exact word-for-word translation as it alters the original instrument and its psychometric properties but is closer to the construct “depression.” Translated instruments normed originally on one population must be normed on a population for whom the instrument was translated. Finally, the underlying construct being measured may manifest itself differently culturally.

### 2.3 Arabic versions of the PTGI

The PTGI-K and the PTGI-T were translated, back-translated (English-Arabic-English) and normed in Gaza. The PTGI-K was translated and back-translated independently by three mental health professionals before reaching a consensus translation in terms of cultural content validity. A fourth mental health professional back-translated this version for both linguistic and cultural accuracy. Minor changes were recommended by the original PTGI author upon revising the penultimate version. The PTGI-K was then validated with 132 Palestinian adults living in Gaza. Alternatively, the PTGI-T was translated and back-translated by a panel of mental health experts and pilot-tested with 35 nurses. Only one item was adjusted for the final translated measure. The final PTGI-T was then validated among nurses working in Gaza.

As presented in Table 1, both PTGI-K and PTGI-T reported adequate convergent and predictive validity with cumulative trauma, depression (PTGI-K), trauma and resilience (PTGI-T), as well as high internal to moderate reliabilities for the full scale (α = 0.96 and 0.86, respectively). The PTGI-K demonstrated moderate to strong alpha coefficients (0.77–0.87) on the five subscale scores. The test-re-test reliability over two months was adequate (*r* = 0.71) [19]. Using Kira's PTGI with 39middle eastern refugees diagnosed with mental disorders in Australia [9], authors in Ref. [20] reported strong internal reliability on the total score (α = 0.96) [20]. Total PTGI-K scores were significantly and inversely related to psychological morbidity (*r* = 0.39). The PTGI-T was subsequently employed in a study with 381 university students living in Gaza and reported a similar reliability score (a = 0.86). Nevertheless, 274 nurses in Gaza were part of a study on trauma and post-traumatic growth using the PTGI-T and reported a higher internal reliability score of 0.94 [40].

### 2.4 Factor structure validity

No conclusive and repeatable evidence was observed for the five-factor structural consistency, which is consistent with the findings reported by researchers in the US. Authors in Ref. [19] used “confirmatory factor analysis (CFA)” to assess the validity of five-factor, three-factor, two-factor, and unitary-factor models. Despite all models having at least a “somewhat satisfactory” fit, only the two-factor model reflected the best fit (p. 129). The two factors were referred to as “internal growth” and “relational growth” with a corresponding subscale internal reliability of 0.95 and 0.84, respectively.

Davey, Heard, and Lennings in Ref. [20] applied the PTGI-K among 40 middle eastern refugee adults diagnosed with mental disorders living in Australia and found a significant negative correlation between total scores and an Arabic version of the “Impact of Events Scale-Revised” (*r* = −0.40). These findings support the assertion of adequate convergent validity reported by Ref. [9]. Meanwhile, the EFA performed by authors in Ref. [20] resulted in four factors rather than two but the CFA was not performed to assess the model fit due to the small sample size.

The two studies found using the PTGI-T did not perform construct validity, instead, the total scores and the original five subscales (mentioned earlier) scores were analyzed. Both the PTGI-K and PTGI-T were normed with people in Gaza, comprising the general population and both nurses and university students living and working in the country. Before conducting the study with Jordanian university students, the two versions of PTGI were studied and the researchers concluded that each of the items could be translated to develop a stronger Arabic version of the PTGI.

Independently, the current research translated and back-translated the PTGI into Arabic and studied the psychometric properties of a large sample of Jordanian college students (PTGI-M). Notably, Palestinian and Jordanian Arabic is classified as Levantine Arabic and considered virtually synonymous, whereby the three measures should be linguistically equivalent. In the present study, a focus group discussion was conducted with nine senior students, three graduate students, and two instructors who are specialised in Counselling and English. The purpose of the discussion was to address the meaningful differences between the items among the three translated scales (PTGI-K, PTGI-T, and PTGI-M). The focus group discussion revealed different opinions for 11 items out of the 21 items in the translation, as well as the PTGI-K and PTGI-T (see Table 1). Specifically, items 1, 2, 4, 5, 8, 9, 11, 12, 15, and 16 were translated differently among the three Arabic versions. The present study established its translation and utilised the translation and the PTGI-M. Next, the psychometric properties were studied by investigating Jordanian university students who had experienced some form of trauma.

The findings from the focus group discussion were thoroughly assessed. Item one in PTGI-K addresses that a person changes priorities generally in life, whereas PTGI-T leads to conclusive changes after the war. In this case, the translation in PTGI-M seems better since it is a common culture that the Jordanian community tends to be specific about their life priorities. Hence, this indicates that item one in PTGI-M represents a serious individual concern. Meanwhile, the different meaning in item two was between the PTGI-K and PTGI-M. The results were consistent as the translation for the current study illustrates that individuals tend to develop a greater appreciation for the value of life. Furthermore, the Jordanian community will be able to understand item 2 in PTGI-K, which implies that individuals appreciate themselves. However, PTGI-M and PTGI-T shared similar translations for item two.

On another note, item four for PTGI-K indicates that a person is more dependent on themselves (independent), whereas PTGI-T implies that the person is too trusting in one's self. Meanwhile, PTGI-M shows that a person is more self-resilience. Both PTGI-M and PTGI-K illustrate a similar meaning for item eight “I have a greater sense of closeness to others”, whereas item eight for PTGI-T was translated as “I am close to others”, which will slightly change the actual PTGI-meaning.

Interestingly, item 12 was translated in three different ways. In this case, the PTGI –K shows that an individual is better at accepting reality while the item in PTGI- T represents that an individual is better at accepting the latest life event after the war. Meanwhile, item 12 in PTGI-M expresses that an individual is better at accepting the way life goes; hence, this is the closest meaning to item 12 in the original PTGI. In item 15, the word “compassion” was translated into three different Arabic words as follows: PTGI-K (Feeling, احساس), PTGI-T (Love and emotionحب وعاطفة), and PTGI-M (Empathy, (تعاطف). On a final note, item 16 in PTGI-T and PTGI-K describes that an individual is putting better effort into establishing a relationship with others. Nonetheless, item 16 in PTGI-M suggests that an individual is putting more effort into personal relationships, which further reflects a deeper individualistic concern for the test taker.

The current study presumed that stress, depression, and anxiety are significant determinants of the extent to which individuals would view PTG by experiencing pressures in life (i.e., stressors). It was also anticipated that PTGI would have a significant negative relationship with the “Taylor Manifest Anxiety Scale (TMAS), Beck Depression Inventory (BDI), and Perceived Stress Scale (PSS)”. Furthermore, it may also appear feasible to acquire significant positive relationships and differences in individual attitudes of students, life skills, and personality attributes regarding PTGI. Satisfaction with life (SWL) and the “Rosenberg Self-Esteem Scale (RSES)” was used to substantiate the concurrent validity of the PTGI scale.

## 3 Instrumentations

The survey packet included a cover letter as informed consent, a demographic section, and the Arabic versions of the PTGI-M, TMAS, PSS, BDI, and the SWL scale. Each scale was computed by summing the scores for all statements or items responded to by the participants, followed by interpreting the score against each scale.

### 3.1 Post-traumatic growth Inventory-M (PTGI-M)

The original PTGI was translated into Arabic by three professional and academic bilingual translators independently and sequentially with the same Likert scale. A translation-back-translation method [41] was conducted to maximize the equivalency upon comparing the three translations. Another two independent bilingual translators blindly back-translated the Arabic version into English. Three additional academics compared the original scale and the two back-translated versions for both literal and cultural-linguistic fluency. Pilot testing of the PTGI-M was performed among 39 university students to estimate its readability and comprehension. No changes were made upon completing the pilot test.

### 3.2 Beck depression inventory (BDI)”

“Beck Depression Inventory (BDI)” is an instrument comprising 21 self-report items that are commonly used in measuring depression. The items are presented with responses ranging from zero to three, representing the absence and severity of depression symptoms. The total scale scores range from zero to 63, where zero to nine represents no or minimal depression, 10 to 18 is mild, 19 to 29 is moderate and 30 to 63 is severe [42]. For this study, the Arabic version of BDI was employed as an indicator of participants’ depression levels. The Arabic version has been tested for psychometric properties and validated among Jordanian university students [43].

### 3.3 “Taylor's Manifest Anxiety Scale (TMAS)”

“Taylor's Manifest Anxiety Scale (TMAS)” is a 50-item scale used as a general measurement of anxiety and as a personality trait rather than a clinical disorder. Participants respond to TAMAS with a True or False for each item. Higher total scores indicate higher adherence to anxiety traits [44]. The Arabic version of TMAS was used in this study. The overall scores range from zero to 16 (absence of anxiety), 17 to 24 (mild anxiety), 25 to 35 (moderate anxiety), and greater than or equal to 36 (severe anxiety). Suleiman and Abdulla in Ref. [45] reported acceptable psychometric characteristics and cultural equivalence of the Arabic version of the TMAS.

### 3.4 “Perceived stress scale (PSS)”

The Perceived Stress Scale is a paper-and-pencil, comprising 14 items that are presented with a five-point Likert-like scale (0 = never and 4 = always) with a total score ranging from zero to five [46]. Higher scores correlate with greater perceived stress. Previous authors have demonstrated that the PSS has adequate psychometric properties [46,47]. The Arabic version of the PSS validated on Jordanians also depicted adequate psychometric properties [48,49].

### 3.5 Satisfaction with life (SWL) scale

The SWL scale was designed to measure individuals’ subjective judgement of life satisfaction [50]. The instrument is a self-report and paper-and-pencil version, comprising five items that are presented using a seven-point Likert-like scale (1 = strongly disagree to 7 = strongly agree). Higher total scores represent greater subjective well-being. The Arabic version of the SWL [51] was employed in this study. Using University students as a norm group, the study reported acceptable to moderate internal and test-retest reliability coefficients of 0.79 and 0.83, respectively and content validity (CV) with related scales.

### 3.6 “The Rosenberg Self-Esteem Scale (RSES)”

The RSES is a widely used 10-item self-report instrument. Participants respond on a four-point Likert-like scale ranging from strongly disagree (score 0) to strongly agree (score 3), and the possible total score ranges from zero to 30. A high total score indicates greater self-esteem [52], and the scale possesses good psychometric properties with a coefficient alpha of 0.89 and test-re-test (r) of 0.83. A previous study also demonstrated good content and construct validity [53]. The Arabic version of RSES [54] was used in the present study.

## 4 Findings

This section presents the empirical findings of this study. All statistical analyses were conducted using SPSS and Smart PLS. Participants' demographic traits and preliminary analysis (missing values, outliers, and data normality) were performed using descriptive statistics. Bivariate correlations between all variables were computed using Pearson's correlation. Test-retest reliability tests and Cronbach's alpha coefficient were conducted to evaluate the “internal consistency” for over three weeks. Confirmatory factor analysis (CFA) was then used to examine the convergent and discriminant validity of the variables.

### 4.1 Demographics

Undergraduate students (N = 446; 57 males, 389 females) enrolled in educational sciences courses at a large university in Jordan were recruited to complete a paper-and-pencil survey packet. A total of 29 students (six males and 23 females) who were identified to have never experienced trauma were excluded from the study before data analysis. Additionally, data from 11 of the 29 students were considered unsuitable for further analysis since at least nine items from the PTGI-M were missing for these subjects. Hence, the dataset from 417 students was subjected to CFA. The students’ (51 males; 366 females) ages ranged from 18 to 42 years old (mean = 21.11, standard deviation [SD] = 2.60), with 93% between 18 and 23 years old. A higher proportion of the participants were seniors (45.3%), single (91.6%), and Muslims (94.9%).

Of the 417 university student participants, only 96 (23.1%) of them identified trauma exposure, while more than one-third of them (76.9%; n = 321) selected “other.” Of the 321 students who selected “other,” only 60% posited that they experienced trauma, including relationship breakup, separation, parental divorce (n = 112; 34.8%), school failure or school stress (n = 48; 14.9%), and bereavement (n = 48; 14.9%). The remaining 96 ticked “serious accidents” (9.13%), “non-sexual assault” (8.41%), “life-threatening illness” (1.9%), “natural disaster” (1.2%), “sexual assault” (0.7%), “torture"(0.7%), “military combat” (0.5%), and “imprisonment” (0.2%). Over half of the participants had experienced trauma in the last three years.

#### 4.1.1 Data screening

Data screening should be performed on unprocessed data before progressing with statistical evaluation to ensure data accuracy. This process is also important to ensure that the gathered data are sufficient to continue with the statistical analyses.

### 4.2 Missing values treatment

Various methods are available to find solutions for missing values in a dataset. Earlier studies recommended replacing missing values with the mean as a simple method of addressing the issue when missing values account for 5.0% or less of the whole data [55]. In the current study, the missing values were replaced using the “mean replacement method” as the missing data were less than 5%. A total of 20 values were observed to be missing in the dataset gathered in this study (Table 2).

**Table 2:** “Missing values”.

Constructs	“Missing Values”
“Relating to Others”	8
“New Possibilities”	5
“Personal Strength”	3
“Spiritual Change”	3
“Appreciation of Life”	1
**Total**	**20**

#### 4.2.1 Multivariate outliers

Outliers are described as “observations that are inconsistent with the rest of the data [56]. Outliers are found to disprove the effect of several values on the average values of items [57]. There are various approaches used in detecting extreme values in data. The current study applied the “Mahalanobis distance statistical analysis” to identify the outliers. This technique can detect observations that are positioned away from the mean values [58]. Therefore, this study employed the “Mahalanobis distance statistical analysis” and no extreme value/outlier was observed.

### 4.3 Statistical assumptions

The present study employed “Smart PLS3” for the statistical analysis. It is important to refer to a few fundamental assumptions of data normality and collinearity related to the variables to validate the results and deal with the occurrence of errors [58].

### 4.4 Multicollinearity

It is essential to evaluate the multicollinearity before the assessment of the model. Table 3 illustrates that the VIF values for all the regressors were less than 5 (VIF <5) as recommended by Ref. [59]. Thus, the dataset was free from multicollinearity issues.

**Table 3:** Multicollinearity.

Constructs	VIF
“Relating to Others”	1.544
“New Possibilities”	1.14
“Personal Strength”	3.348
“Spiritual Change”	1.045
“Appreciation of Life”	3.356

### 4.5 Data normality

It is important to evaluate the data normality before employing inferential statistical techniques [60]. Based on the recommendation in Ref. [61], the present study examined the data normality using the “Skewness, Kurtosis and histogram plots”. Resultantly, the data were not normally distributed. Nevertheless, no extremely non-normal data were detected. According to Ref. [62], data normality is not an issue in PLS-SEM since it is a non-parametric method that does not need the data to be normally dispersed. The current study thus progressed with the subsequent analysis using PLS-SEM.

### 4.6 Confirmatory factor analysis (measurement model assessment)

The study used PLS-SEM which involved a two-staged process, namely, “measurement model and structural model assessments” [59,63]. The measurement model evaluates the relationship between the variables and items or indicators [64]. According [63], the measurement model is evaluated based on convergent validity and discriminant validity. The reflective measurement model is assessed based on the validity and reliability of the latent variables [62]. The present study employed the CFA to assess the measurement model by probing the association between the indicators/items and their relevant variables. The CFA was also performed to evaluate and verify the “internal consistency, convergent validity and discriminant validity” of every scale. Specifically, the reliability was evaluated using composite reliability (CR) while convergent validity and discriminant validity were applied to measure the construct validity.

#### 4.6.1 Composite reliability (CR)

**Fig. 1:**
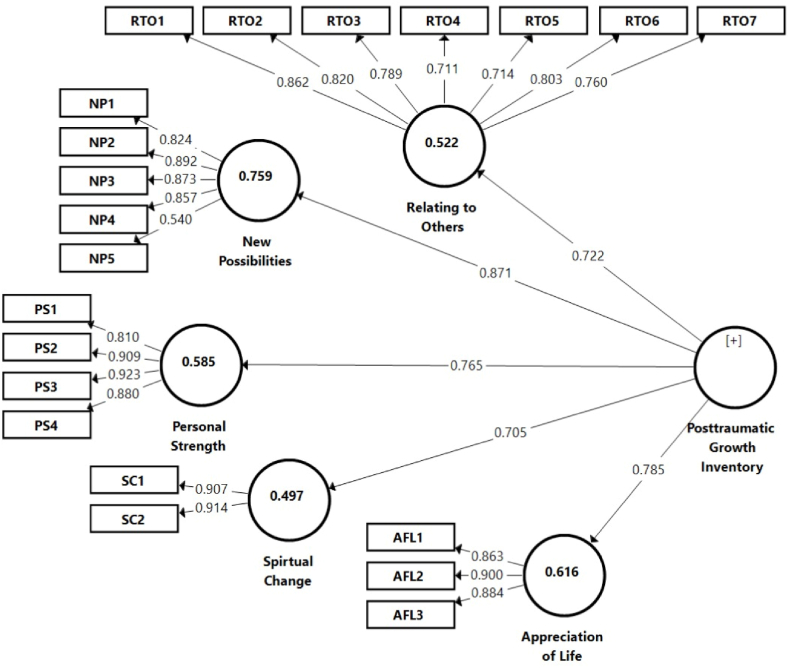
Confirmatory factor analysis.

Composite reliability (CR) accomplishes the same task as “Cronbach's alpha”, but the former offers a more robust method of assessing internal consistency reliability [65,66]. In this study, the CR was measured to evaluate the internal of the constructs. Table 4 reveals that all the items were loaded on their respective constructs. As shown in Fig. 1, all the loadings were more than the suggested threshold of 0.50. Moreover, items with lower loadings were eliminated to obtain the required threshold value of the CR. Likewise, all the variables recorded internal consistency values that were within e satisfactory range upon deleting some items in the constructs. Table 4 denotes that the CR values for all the variables ranged from 0.88 to 0.93, which is higher than the minimum value of 0.70 [67]. The findings illustrated that all the variables had a high level of inter-item consistency.

**Table 4:** Reliability and convergent validity.

1st Order Constructs	2nd Order Construct	Items	Loadings	Alpha	CR	AVE
Relating to Others		RTO1	0.86	0.89	0.92	0.61
		RTO2	0.82			
		RTO3	0.78			
		RTO4	0.71			
		RTO5	0.71			
		RTO6	0.80			
		RTO7	0.76			
New Possibilities		NP1	0.82	0.86	0.90	0.65
		P2	0.89			
		NP3	0.87			
		NP4	0.85			
		NP5	0.54			
Personal Strength		PS1	0.81	0.90	0.93	0.78
		PS2	0.91			
		PS3	0.92			
		PS4	0.88			
Spiritual change		SC1	0.91	0.79	0.91	0.83
		SC2	0.91			
Appreciation of Life		AFL1	0.86	0.86	0.91	0.78
		AFL2	0.90			
		AFL3	0.88			
	Post-traumatic Growth Inventory	RTO	0.72	0.82	0.88	0.60
		NP	0.87			
		PS	0.76			
		SC	0.70			
		AFL	0.785

#### 4.6.2 Construct validity

Construct validity assesses the extent that the results obtained using a measure fit the theories around the designed test [68] Convergent validity and discriminant validity are the two major categories of construct validity [69].

### 4.7 Convergent validity

According to Ref. [67], the “average variance extract” (AVE) is applied to validate the convergent validity [70]. The AVE of constructs should be greater than 0.50 for establishing sufficient convergent validity [62]. According to Table 4, all the AVE values were in the satisfactory range. The AVE values were higher than 0.50 and ranged from 0.59 to 0.83, which indicated adequate convergent validity. The AVE values were higher than 0.50, reflecting that the latent constructs elucidated more than half of the variation of their respective indicators. Hence, the convergent validity for all the variables was validated in this study.

#### 4.7.1 Discriminant validity

Discriminant validity refers to the degree to which a variable is distinct from other variables [67]. This study engaged two methods to evaluate discriminant validity; Fornell and Larcker Criterion [71], and the heterotrait-monotrait ratio [72].

### 4.8 Fornell & Larcker Criterion

The discriminant validity was assessed using the “Fornell & Larcker Criterion”, and the “square root of the AVE” for all the variables was applied and matched compared to the correlation values of the other variables [71]. The square root of the AVE coefficients was demonstrated in the correlation matrix along the diagonal. The discriminant validity is confirmed when the square root of the AVE is higher than the square correlation estimates [58]. Table 5 depicts that the square root of AVE values exceeds the correlation of all variables. All diagonal elements were larger than the off-diagonal values in the subsequent rows and columns, which confirms the acceptable discriminant validity of all the variables.

**Table 5:** Fornell-larcker criterion.

	AFL	NP	PS	PGI	RTO	SC
AFL	**0.88**					
NP	0.66	**0.81**				
PS	0.54	0.56	**0.88**			
PGI	0.79	0.77	0.76	**0.64**		
RTO	0.43	0.53	0.28	0.52	**0.78**	
SC	0.44	0.53	0.63	0.40	0.35	**0.91**

**Note: “**AFL- Appreciation of Life; NP- New Possibilities; PS- Personal Strength; PGI- Posttraumatic Growth Inventory; RTO- Relating to Others; SC- Spiritual Change.

### 4.9 Cross-loadings

The cross-loadings of the indicators were also assessed in this study. According to Ref. [67], the loading values should be 0.50 or higher and the items with the lowest factor loadings should be eliminated. All items of a variable should be considered loaded on their respective variables [62]. Table 6 illustrates that the loadings of all items were higher than the cross-loadings of the other variables. All the indicators were loaded on their respective variables and no cross-loading was present in the various items.

**Table 6:** Cross loadings.

	AFL	NP	PS	RTO	SC
AFL1	**0.863**	0.580	0.404	0.373	0.363
AFL2	**0.900**	0.580	0.505	0.409	0.419
AFL3	**0.884**	0.582	0.536	0.364	0.387
NP1	0.580	**0.824**	0.411	0.444	0.454
NP2	0.553	**0.892**	0.443	0.475	0.382
NP3	0.472	**0.873**	0.388	0.445	0.369
NP4	0.563	**0.857**	0.427	0.496	0.345
NP5	0.464	**0.540**	0.618	0.269	0.622
PS1	0.442	0.449	**0.81**	0.225	0.421
PS2	0.544	0.513	**0.909**	0.272	0.582
PS3	0.471	0.528	**0.923**	0.260	0.597
PS4	0.470	0.498	**0.880**	0.256	0.624
RTO1	0.429	0.460	0.254	**0.862**	0.278
RTO2	0.408	0.456	0.250	**0.820**	0.230
RTO3	0.242	0.364	0.201	**0.789**	0.315
RTO4	0.291	0.455	0.260	**0.711**	0.304
RTO5	0.395	0.449	0.222	**0.714**	0.331
RTO6	0.357	0.391	0.203	**0.803**	0.198
RTO7	0.205	0.329	0.171	**0.760**	0.278
SC1	0.430	0.453	0.597	0.286	**0.907**
SC2	0.376	0.519	0.560	0.354	**0.914**

**Note:** “BDA-Big Data Analytics, CC-Cloud Computing, AI-Artificial Intelligence, R-Robotics, IOT-Internet of Things, SOP-Sustainable organization Performance”.

### 4.10 Heterotrait-monotrait ratio”

The latest criterion to assess discriminant validity for “structural equation modelling” was presented by Ref. [72]. The researchers argued that the Fornell-Larcker criterion and cross-loadings did not detect the discriminant validity in several research situations. Authors in Ref. [72] proposed an alternative method - “the heterotrait-monotrait ratio of correlations”, which is based on the “multitrait-multimethod matrix” to measure the discriminant validity. This study worked with the approach proposed by Clark and colleagues (2011) in which the HTMT ratio should be less than 0.85 or 0.90 [72,73]. A problem of discriminant validity arises when the HTMT ratio is higher than the above-mentioned thresholds. Table 7 presents all the HTMT ratio values for all understudy variables. All the variables recorded HTMT values less than 0.90. As described by Ref. [73], these findings indicated all the variables and constructs were free from discriminant validity issues.

**Table 7:** HTMT ratio.

	AFL	NP	PS	PGI	RTO	SC
AFL						
NP	0.77					
PS	0.62	0.65				
PGI	0.86	0.97	0.80			
RTO	0.49	0.61	0.32	0.84		
SC	0.53	0.66	0.74	0.80	0.41	

### 4.11 Reliability analysis

Table 8 denoted that the total score of the translated Arabic PTGI in this study demonstrated a high Cronbach's alpha coefficient (α = 0.97), thereby indicating strong reliability. Likewise, a liability was observed for the BDI (α = 0.87), TMAS (α = 0.86), and RSES (α = 0.89). Concurrently, the internal reliability for PSS (α = 0.80) and SWL (α = 0.79) from the sample of Jordanian university student scores was moderate. The test-retest reliability values (*r*) at two-time points were 0.72 and 0.83 respectively.

### 4.12 Concurrent validity

The PTGI-M scores correlated negatively with the total scores on mental health as measured by the BDI (*r* = −0.90), the PSS (*r* = −0.86), and the TMAS (*r* = −0.85). As expected, the PTGI-M scores were positively correlated to students’ life satisfaction (*r* = 0.90) and self-esteem (*r* = 0.84) lending credence to the CV of the PTGI-M scores (see Table 8). Based on the factor correlation matrix results, there are compelling reasons to maintain the non-orthogonal rotation method as some correlations exceeded the minimum value of 0.32 [64].

**Table 8:** “Summary of Means, Standard Deviations, Intercorrelations, and Cronbach's alpha for all constructs with the dependent variable” (n = 417).

Variable	(PTG)	(BDI)	(TMAS)	(PSS)	(SWL)	(RSES)	M	SD	(α)	r1	r2
PTG	–	−0.90**	−0.85**	−0.86**	0.89**	0.84**	76.11	18.68	0.83	0.71	0.83
BDI	–	–	0.93**	0.89**	−0.90**	−0.82**	16.13	10.73	0.87		
TMAS	–	–	–	0.91**	−0.87**	−0.79**	17.99	9.36	0.86		
PSS	–	–	–	–	−0.88**	−0.79**	19.20	10.1	0.80		
SWL	–	–	–	–	–	0.83**	21.70	6.50	0.79		
RSES	–	–	–	–	–	–	24.40	7.10	0.89		
**p < 0.01											

Note- “PTG = Post-traumatic Growth Inventory, BDI = Beck Depression Inventory, TMAS = Taylor Manifest Anxiety Scale, PSS = Perceived Stress Scale, SWL = Satisfaction with Life, RSES = Rosenberg Self-Esteem Scale, α = Cronbach's alpha, M = mean, SD = standard deviation, *r*_*1*_*and r*_*2*_*=* test-retest reliability at first week and three weeks, **p < 0.01″

Cronbach's alpha values for the PTGI-M total scale and subscale scores.

Table 9 depicts that the PTGI-M total scale indicated strong internal consistency (Total, α = 0.82), as well as the subscales: Relating to others (α = 0.89), New possibilities, (α = 0.857), Appreciation of life (α = 0.85), and Personal Strength (α = 0.90). Meanwhile, for all scales and subscales of the PTGI-M, females reported higher growth scores than males.

**Table 9:** “Sample Means, Standard Deviations, and Cronbach's alpha on PTGI-M for the studied population (N = 417)”.

			PTGI-M						
			All (N = 417)		Females (n = 366)		Males (n = 51		
PTGI Subscale	Item	**Score Range**	**M**	SD	**M**	**SD**	M	**SD**	α
**Relating to Others**	7	0–35	17.11	4.88	18.17	4.81	16.84	5.21	0.89
**New Possibilities**	5	0–25	17.77	4.90	18.50	4.50	15.98	6.71	0.85
**Personal Strength**	4	0–20	11.11	2.80	11.25	2.69	10.21	3.27	0.90
**Spiritual Change**	2	0–10	7.69	1.94	7.75	1.90	7.33	2.13	0.90
**Appreciation of Life**	3	0–15	10.75	2.92	10.88	2.81	9.91	3.41	0.85
									
**Total PTGI**	**21**	**0–105**	**76.11**	**18.68**	**77.80**	**17.87**	**70**	**22.3**	**0.97**

**Note:** PTGI = “Post-traumatic Growth Inventory, α = Cronbach's alpha, SD = standard deviation, *M* = mean”.

## 5 Comparisons of reliability tests and outcomes of PTGI-K, PTGI-T, and original PTGI Total Score and Subscale Scores

The translated Arabic PTGI recorded a high reliability for its total score (α = 0.97), which is similar to the reports by authors in Ref. [9]. In contrast to Kira and his colleagues [9], the original subscales of the translated Arabic PTGI were mostly strong. Excluding Spiritual Change with two items, the PTGI-M total score and sub-score means were lower than the corresponding scores reported by authors in Ref. [8] (See Table 2, Table 6). In the original norm group for the PTGI, females demonstrated higher growth scores for all the scales and subscales of the PTGI-M compared to males (Table 10).

**Table 10:** “Reliability tests and outcomes of PTGI-K, PTGI-T, and original PTGI Total Score and Subscale Scores

		Original study							PTGI-T			PTGI-K		
		Tedeschi & Calhoun (1996)							Thabet et al. (2015)			Kira et al. (2012)		
		All (N = 604)	Females (n = 405)			Males (n = 199)			All (N = −)			Adults(N = 132)		
**PTGI Subscale**	Item	M	SD	M	SD	M	SD	Α	M	SD	α	M	SD	α
**Relating to Others**	7	26.49	–	29.68	–	23.30	–	0.85	18.15	5.11	–	23.89	8.87	0.87
**New Possibilities**	5	19.65	–	20.94	–	18.35	–	0.84	12.25	3.74	–	18.43	7.15	0.86
**Personal Strength**	4	16.60	–	17.90	–	15.30	–	0.67	10.62	3.14	–	15.30	5.81	0.86
**Spiritual Change**	2	6.63	–	8.29	–	4.96	–	0.72	6.82	1.53	–	8.01	3.28	0.86
**Appreciation of Life**	3	12.58	–	13.45	–	11.70	–	0.85	7.17	2.49	–	10.83	4.23	0.77
**Total PTGI**	**21**	81.94	–	90.26	–	73.61	–	0.90	67.34	13.42	.86	55.54	26.84	**0.96**

“Reliability tests and outcomes of PTGI-K, PTGI-T, and original PTGI Total Score and Subscale Scores.

## 6 Discussion

The current study investigated the psychometric properties of an Arabic version of the PTGI. The research reported that PTGI-M has good psychometric properties, which were depicted by the high reliability of the main inventory and its subscale. The reliability of the 21-item PTGI was 0.880, ranging from 0.88 to 0.95. Hence, a significant internal consistency was detected for the five factors, which aligned with the original scale in Ref. [8] and other studies [74,75] The test-retest reliability (*r*) over three weeks ranged from 0.71 to 0.83, which is consistent with the reports by authors in Ref. [8] where a value of 0.71 was obtained in their test-retest reliability for two months. The PTGI dimensionality was also assessed in this study. Meanwhile, the present study is the first attempt to elucidate the PTGI dimensionality in a Jordanian population using a relatively large sample size of undergraduate students.

This study employed all the necessary steps involved in CFA by applying Smart PLS. Convergent validity was established based on the CFA and AVE. Fornell Lacker and HTMT ratio were also applied to evaluate the discriminant validity of the constructs. Resultantly, both convergent and discriminant validity was established for the Arabic version of the PTGI. Furthermore, the factor loadings for all 21 items of the PTGI ranged from 0.71 to 0.92, suggesting that all the items are suitable indicators of their corresponding constructs. These scores depict the good construct validity of the PTGI factor structure, thereby supporting its multidimensional measure and characteristics.

## 7 Implication

The growing globalisation and influence of Western nations on other countries have placed ethical, educational, and political pressure on counsellors to ensure the implementation of evidence-based and culturally sensitive counselling principles across different cultures. According to “The Nations High Commissioner for Refugees in Ref. [2], United Global wars, violence, and abuse of human rights have also contributed to the increasing population of asylum-seekers and refugees absconding to developed nations. Furthermore, the same report reflects that most of those fleeing to other countries have suffered from traumatic circumstances and are often referred to seek assistance from mental health services. In conclusion, the development research and supporting theories on PTG remain unclear to the degree to which the issues associated with cultural prejudice prevail. Accordingly, more research is warranted to address these issues.

Several earlier PTGI studies have reported different numbers and a variety of factors [76]. Two factors emerged from the present study, which was similar to the findings conducted among Palestinians by Ref. [9]. In contrast, authors in Ref. [20] suggested five components in a study undertaken among Arabic-speaking refugees in Australia. The fact that Arabic-speaking people are not a monocultural group might explain some of these discrepancies. Despite the similarity in the formal written Arabic, three different dialects of Arabic are spoken throughout the world known as Egyptian, Gulf, and Levantine Arabic. Palestinians and Jordanians usually speak Levantine Arabic. In comparison, the refugees studied by authors in Ref. [20] were from different areas (the majority from Iraq, where Egyptian Arabic is spoken) and they might have spoken different dialects. Language is inherently cultural, and it would be interesting to consider how PTG is conceptualised among Arabic-speaking populations. More studies are required to investigate the validity and consistency of the PTGI-M factors in different cultural backgrounds and to determine scale cultural biases [35]. Future analyses are also encouraged to consider both the subscale and total scores of the PTGI to understand the complexity of experiencing post-traumatic growth.

## 8 Conclusion

The study assessed the Arabic-translated version of the PTGI by focusing on the validity of the scale in different languages and contexts. It introduced an “Arabic version of the Post-Traumatic Growth Inventory (PTGI-M)”. Both convergent and discriminant validity was established for the Arabic version of this scale. The current findings revealed that the Arabic translation of PTGI-M is an important measure and could be employed to screen the overall growth of the Arabic-speaking community with a traumatic experience. Nevertheless, Arabic PTGI needs to be explored to investigate the factor components.

## 9 Limitations

Several limitations were inherent in this study. Authors in Ref. [77] indicated that the incidence ratios of trauma among students were similar to those observed in the general community. However, the present study enrolled undergraduate students mainly from a single university in Jordan. Meanwhile, there are currently a total of 36 Jordanian universities comprising mainly female and unmarried (single) individuals. These differences limit the general ability of the present findings to other universities in Jordan. Similarly, these findings may be different compared to other populations with different demographic attributes. Therefore, future research should be considered if the present version of the Arabic PTGI attains similar psychometric properties in other sample populations.

Another limitation of the study relates to factor analysis design. While the total respondents who participated in this study met the least number considered acceptable, a higher number of participants and sample sizes are suggested for future research [78]. Therefore, the present findings need to be viewed and interpreted carefully. Another limitation may relate to the explicitness or clarity surrounding the items (units) in this study. Given the differences in participants’ background, culture, language, and educational upbringing, their level of understanding of the statements/questions about their background and the consequences of culturally-based social desirability on their responses were challenging to gauge. Nonetheless, this limitation was addressed through the selection of translators and the translation process. Moreover, this study used cross-sectional research design that is another limitation that does not allow to interpret the associations between the constructs in a causal manner. Future studies may adopt longitudinal design with time lags data to have more accurate findings.

## Declarations

### Author contribution statement

Mais AL-Nasa'h: Conceived and designed the experiments; Performed the experiments; Analyzed and interpreted the data; Contributed reagents, materials, analysis tools or data; Wrote the paper.Kimberly Asner-Self: Conceived and designed the experiments; Contributed reagents, materials, analysis tools or data; Wrote the paper.Hassan Al Omari: Performed the experiments; Analyzed and interpreted the data; Wrote the paper.Amani Qashmer: Contributed reagents, materials, analysis tools or data; Wrote the paper.Mohammad Alkhawaldeh: Analyzed and interpreted the data; Wrote the paper.

### Funding statement

This research did not receive any specific grant from funding agencies in the public, commercial, or not-for-profit sectors.

### Data availability statement

Data will be made available on request.

### Declaration of interest's statement

The authors declare no competing interests.

## Figures and Tables

**Table:** 

Checklist		Response
An indicative list of prerequisites		
1	We obtain the required permission from the author's email who owns the publication right before conducting this translation and testing it to the new culture in Jordan.	✓
2	It was ensured that the definition and content within the amount of the hypothetical configuration construct the measurement by test, and the content of the items in the communities to which the test will be transferred, are sufficient for the purpose according to the intent of the use of the test scores.	✓
3	We tried to Minimize the cultural and linguistic differences unrelated to the intended uses of the test in the Jordan culture to which it was transmitted.	✓
**Guidance List for Test Development**		
1	Ensuring that translation processes and test transfer to a new culture take into account linguistic, psychological and cultural differences in target communities through the selection of experts with appropriate experience	✓
2	Using appropriate translation designs and procedures to maximize the convenience of the processed transfer of the test to a new culture in the target communities.	✓
3	Providing evidence that test instructions and item content have a similar meaning to all target communities.	✓
4	Providing evidence that item formats, grading metrics, registration categories and test instructions, methods of application, and other procedures are appropriate for all target communities.	✓
5	Collect survey data from the new test version(pilt); so that items can be analyzed, and assess consistency and study validity (on a small sample) so that any necessary revisions can be made for the new test version.	✓
**Checklist**		
**1**	Choosing a sample with characteristics related to the intended use of the test and sufficient size for the empirical analyses you will perform.	✓
**2**	Providing relevant statistical evidence on structural equivalence, methodological equivalence, and equivalence on Item level for all target communities.	✓
**3**	Providing evidence supporting standards, consistency, and truthfulness of the codified version of the test in the community Target.	✓
**4**	Using appropriate equation design and data analysis procedures when linking metrics Scores from test versions in different languages.	✓
**Test Administration and Management Guidance List**		
	Preparation of test management materials and instructions for its administration to minimize any problems related to culture and the language that may result from the procedures of administrating the test and the response patterns, which can affect the validity of conclusions derived from grades.	✓
	Determine which test conditions should be closely followed when administrating the test to all target communities.	✓
**Indicative List for Monitoring and Interpreting Grades**		
	Interpret any differences in group scores by reference to all related available information.	**N/A**
	Compare scores across communities only after determining the level of equivalence on the scale where grades are recorded.	**N/A**
**An indicative list of reports and documentation**		
	provide technical feedback for any changes, including a list of the evidence obtained, to support parity, when the test is transferred for use in another community.	**N/A**
	Provide documentation to test users that will support properly used practice transferred testing with people in the context of the new society.	✓
