# Supplementary material for: “Psychometric properties of the Arabic version of the post-traumatic growth inventory with university students in Jordan
Source: Heliyon. 2023 Mar 9;9(3):e14211. doi: 10.1016/j.heliyon.2023.e14211 (PMC10010994; doi:10.1016/j.heliyon.2023.e14211)
Supplement: Multimedia component 1 [file mmc1.docx]

Table 2

*“Missing Values”*

| **Constructs** | **“Missing Values”** |
| --- | --- |
| “Relating to Others” | 8 |
| “New Possibilities” | 5 |
| “Personal Strength” | 3 |
| “Spiritual Change” | 3 |
| “Appreciation of Life” | 1 |
| **Total** | **20** |

Table 3

*Multicollinearity*

| **Constructs** | **VIF** |
| --- | --- |
| “Relating to Others” | 1.544 |
| “New Possibilities” | 1.14 |
| “Personal Strength” | 3.348 |
| “Spiritual Change” | 1.045 |
| “Appreciation of Life” | 3.356 |

Table 6

*Cross Loadings*

|  | **AFL** | **NP** | **PS** | **RTO** | **SC** |
| --- | --- | --- | --- | --- | --- |
| AFL1 | **0.863** | 0.580 | 0.404 | 0.373 | 0.363 |
| AFL2 | **0.900** | 0.580 | 0.505 | 0.409 | 0.419 |
| AFL3 | **0.884** | 0.582 | 0.536 | 0.364 | 0.387 |
| NP1 | 0.580 | **0.824** | 0.411 | 0.444 | 0.454 |
| NP2 | 0.553 | **0.892** | 0.443 | 0.475 | 0.382 |
| NP3 | 0.472 | **0.873** | 0.388 | 0.445 | 0.369 |
| NP4 | 0.563 | **0.857** | 0.427 | 0.496 | 0.345 |
| NP5 | 0.464 | **0.540** | 0.618 | 0.269 | 0.622 |
| PS1 | 0.442 | 0.449 | **0.81** | 0.225 | 0.421 |
| PS2 | 0.544 | 0.513 | **0.909** | 0.272 | 0.582 |
| PS3 | 0.471 | 0.528 | **0.923** | 0.260 | 0.597 |
| PS4 | 0.470 | 0.498 | **0.880** | 0.256 | 0.624 |
| RTO1 | 0.429 | 0.460 | 0.254 | **0.862** | 0.278 |
| RTO2 | 0.408 | 0.456 | 0.250 | **0.820** | 0.230 |
| RTO3 | 0.242 | 0.364 | 0.201 | **0.789** | 0.315 |
| RTO4 | 0.291 | 0.455 | 0.260 | **0.711** | 0.304 |
| RTO5 | 0.395 | 0.449 | 0.222 | **0.714** | 0.331 |
| RTO6 | 0.357 | 0.391 | 0.203 | **0.803** | 0.198 |
| RTO7 | 0.205 | 0.329 | 0.171 | **0.760** | 0.278 |
| SC1 | 0.430 | 0.453 | 0.597 | 0.286 | **0.907** |
| SC2 | 0.376 | 0.519 | 0.560 | 0.354 | **0.914** |

**Note:** “BDA-Big Data Analytics, CC-Cloud Computing, AI-Artificial Intelligence, R-Robotics, IOT-Internet of Things, SOP-Sustainable organization Performance”

***Table 11***

|  |  | Original study | | | | | | | |  |  | PTGI-T | | | | | |  | PTGI-K | | |
| --- | --- | --- | --- | --- | --- | --- | --- | --- | --- | --- | --- | --- | --- | --- | --- | --- | --- | --- | --- | --- | --- |
|  |  | Tedeschi & Calhoun (1996) | | | | | | | |  | | | Thabet et al. (2015) | | | |  | Kira et al. (2012) | | | |
|  |  | All (N= 604) | | Females  (n = 405) | | | Males  (n =199) | | |  | | | All (N=--) | | | |  | Adults(N=132) | | |  |
| PTGI Subscale | Item | M | SD | | M | SD | M | SD | | Α | | | M | SD | | | α | M | | SD | α |
| Relating to Others | 7 | 26.49 | - | | 29.68 | - | 23.30 | | - | 0.85 | | | 18.15 | | 5.11 | - | | 23.89 | | 8.87 | 0.87 |
| New Possibilities | 5 | 19.65 | - | | 20.94 | - | 18.35 | | - | 0.84 | | | 12.25 | | 3.74 | - | | 18.43 | | 7.15 | 0.86 |
| Personal Strength | 4 | 16.60 | - | | 17.90 | - | 15.30 | | - | 0.67 | | | 10.62 | | 3.14 | - | | 15.30 | | 5.81 | 0.86 |
| Spiritual Change | 2 | 6.63 | - | | 8.29 | - | 4.96 | | - | 0.72 | | | 6.82 | | 1.53 | - | | 8.01 | | 3.28 | 0.86 |
| Appreciation of Life | 3 | 12.58 | - | | 13.45 | - | 11.70 | | - | 0.85 | | | 7.17 | | 2.49 | - | | 10.83 | | 4.23 | 0.77 |
|  |  |  |  | |  |  |  | |  |  | | |  | |  |  | |  | |  |  |
| Total PTGI | 21 | 81.94 | - | | 90.26 | - | 73.61 | | - | 0.90 | | | 67.34 | | 13.42 | .86 | | 55.54 | | 26.84 | 0.96 |

*“Reliability tests and outcomes of PTGI-K, PTGI-T, and original PTGI Total Score and Subscale Scores”*
